# Re-evaluation of blood mercury, lead and cadmium concentrations in the Inuit population of Nunavik (Québec): a cross-sectional study

**DOI:** 10.1186/1476-069X-7-25

**Published:** 2008-06-02

**Authors:** Julie Fontaine, Éric Dewailly, Jean-Louis Benedetti, Daria Pereg, Pierre Ayotte, Serge Déry

**Affiliations:** 1 Unité de recherche en Santé publique, Centre de recherche du CHUL-CHUQ, 2875 boul. Laurier, Bureau 600, Québec (Québec), G1V 2M2, Canada; 2 Institut national de santé publique du Québec, 945, avenue Wolfe, Québec (Québec), G1V 5B3, Canada; 3 Regional Board of Health and Social Services of Nunavik, C.P. 900, Kuujjuaq (Québec), J0M 1C0, Canada

## Abstract

**Background:**

Arctic populations are exposed to mercury, lead and cadmium through their traditional diet. Studies have however shown that cadmium exposure is most often attributable to tobacco smoking. The aim of this study is to examine the trends in mercury, lead and cadmium exposure between 1992 and 2004 in the Inuit population of Nunavik (Northern Québec, Canada) using the data obtained from two broad scale health surveys, and to identify sources of exposure in 2004.

**Methods:**

In 2004, 917 adults aged between 18 and 74 were recruited in the 14 communities of Nunavik to participate to a broad scale health survey. Blood samples were collected and analysed for metals by inductively coupled plasma mass spectrometry, and dietary and life-style characteristics were documented by questionnaires. Results were compared with data obtained in 1992, where 492 people were recruited for a similar survey in the same population.

**Results:**

Mean blood concentration of mercury was 51.2 nmol/L, which represent a 32% decrease (p < 0.001) between 1992 and 2004. Mercury blood concentrations were mainly explained by age (partial r^2 ^= 0.20; p < 0.0001), and the most important source of exposure to mercury was marine mammal meat consumption (partial r^2 ^= 0.04; p < 0.0001). In 2004, mean blood concentration of lead was 0.19 μmol/L and showed a 55% decrease since 1992. No strong associations were observed with any dietary source, and lead concentrations were mainly explained by age (partial r^2 ^= 0.20.; p < 0.001). Blood cadmium concentrations showed a 22% decrease (p < 0.001) between 1992 and 2004. Once stratified according to tobacco use, means varied between 5.3 nmol/L in never-smokers and 40.4 nmol/L in smokers. Blood cadmium concentrations were mainly associated with tobacco smoking (partial r^2 ^= 0.56; p < 0.0001), while consumption of caribou liver and kidney remain a minor source of cadmium exposure among never-smokers.

**Conclusion:**

Important decreases in mercury, lead and cadmium exposure were observed. Mercury decrease could be explained by dietary changes and the ban of lead cartridges use likely contributed to the decrease in lead exposure. Blood cadmium concentrations remain high and, underscoring the need for intensive tobacco smoking prevention campaigns in the Nunavik population.

## Background

Human exposure to environmental contaminants is a well-known phenomenon in the Canadian Arctic. The Inuit of Nunavik are exposed to a plethora of toxic substances that are carried from southern to northern latitudes by oceanic and atmospheric transport and biomagnified in Arctic food webs. As the Inuit traditional diet comprises large amounts of tissues from marine mammals, fish and terrestrial wild game, Inuit are more exposed to metals than populations living in southern regions. Metals of concern include mercury, lead and cadmium.

Mercury is a toxic metal originating from both anthropogenic and natural sources. Despite significant reduction in mercury emissions in Europe and North America, mercury concentrations are still high in the Arctic environment and biota [1]. Although most of the mercury released in the environment is inorganic or elemental, once in the water, it can be transformed into methylmercury (MeHg) by microbial action [2]. This highly toxic form of mercury is accumulated in animal tissues and is biomagnified in the food chain [3]. In the Arctic, the most significant sources of human exposure to MeHg are fish and marine mammal consumption [4-8].

Contaminant exposure from traditional food consumption among Inuit has been estimated during an extensive dietary survey covering Nunavut and Inuvialuit communities in the late 1990s [9]. Data showed that the mean intake levels of mercury were below the tolerable daily intake levels used by Health Canada. Ringed seal kidney had the highest concentration of mercury (14.2 nmol/g) but only contributed 2.5% of total intake during late winter, while ringed seal meat had much lower concentration (2.0 nmol/g) but contributed more (15.3%) to mercury intake. Caribou meat had low mercury concentrations (0.28 nmol/g) but due to its frequent consumption, it was the most important contributor (30.1%) to mercury intake. A recent update in on dietary contaminant exposure done by the same team showed that the contaminants concentrations in 2007 appeared to be similar, with the exception of mercury in ringed seal liver, which was higher, and walrus blubber, which was lower[10].

In adults, MeHg mainly affects the nervous system and is also toxic to the kidney, liver, reproductive organs, and the cardiovascular system [11-13]. Low chronic prenatal exposure similar to that observed in populations exposed through fish consumption may have subtle neurodevelopment consequences [14-16]. In Nunavik, visual information processing [17] and higher tremor amplitude [18] were shown to be related to MeHg exposure in preschool-aged Inuit children. The effects of prenatal exposure to mercury have raised controversy since no detrimental effects were observed in the Seychelles Child Development Study [19], but new preliminary results reveal adverse associations with MeHg when the statistical models were adjusted for nutrient status (fish consumption)[20].

Lead is another toxic metal to which the Inuit may be exposed environmentally. Most of the lead in the environment comes from anthropogenic sources and is carried to the Arctic by atmospheric transport [1]. It has been clearly shown that environmental levels of lead have been decreasing in Arctic regions since the ban of leaded gasoline [1,8]. However, high levels of lead can still be found in Inuit populations in certain Arctic regions due to the past and/or present use of lead shot for hunting wild game [1,5,21]. Hunters can be exposed to lead by inhalation or ingestion of lead dust released by the friction of the shot against the barrel and by the combustion of high-explosive primers that contain lead styphnate[22]. Moreover, consumer of wild game can be exposed to lead by ingestion of whole pellets or fragments embedded in meat or by ingestion of game with biologically incorporated lead (mostly exposed through ingestion of spent shot and fishing sinkers)[22]. In a previous dietary survey in Inuit communities of the Canadian Arctic, the major contributors to lead dietary exposure during late winter were caribou meat (68.4%; 783 ng/g) and Arctic char (21.4%; 1009 ng/g) [9].

In 1999, the use of lead cartridges for hunting migratory birds was banned in Canada, and the public health authorities of Nunavik actively informed the population in order to reduce lead shot use and lead exposure [21]. Dallaire et *al *(2003) [23] analyzed 251 cord blood samples between 1994 and 2001, and results showed a strong decrease in blood concentrations of lead after the ban of lead cartridges (0.20 μmol/L before 1999 compared to 0.12 μmol/L after 1999; *p *< 0.0001).

Environmental exposure to lead can have detrimental neurological effects in children and adults [24-26]. In Nunavik, blood lead concentration in Inuit children was associated with deficits in several fine motor tasks [18] and correlated positively with impulsivity and activity [27]. Plusquellec et *al* (2007) [28] evaluated early behavioural effects of lead in of 169 11-months old Inuit infants and found that cord blood lead concentrations were significantly negatively associated to the direct observational measures of infant attention.

Cadmium is a toxic metal that can cause several health effects [29-32], mainly kidney and bone damage, even in non occupationally exposed populations, and studies indicate health concerns at low dose of exposure either in adults [33-37] or children [38]. Cadmium is released in the arctic environment from both anthropogenic and natural sources and it accumulates in lichen and vegetation [39,40], which is then eaten by caribou and moose [41]. Cadmium accumulates in organs rather than in muscle or fat and it is typically higher in kidneys than in the liver [41], which are part of the traditional diet of several aboriginal people. In Nunavik, caribou meat is the most popular traditional food [42], but no information regarding the importance of caribou kidneys and liver consumption is available. High concentrations of cadmium in kidneys and liver of caribou and moose were reported in the Canadian Arctic [39,41,43,44]. Results from a study conducted in Nunavik (1994–1996) revealed that concentrations of cadmium exceeded tolerance recommendations for human consumption in nearly all caribou kidney samples collected and in 45% of the liver samples [41]. Mean concentration (wet weight) was 7.7 μg/g in kidneys and 1.1 μg/g in liver.

However, previous studies have shown that human exposure to cadmium is most often attributable to tobacco smoking. A study in Canadian Arctic populations as well as three studies in the Inuit population of Nunavik showed that blood cadmium concentrations were mainly associated with tobacco use [45-48], despite the frequent consumption of caribou and moose. Indeed, tobacco can accumulate relatively high concentrations of cadmium in its leaves [49]. Cadmium content of cigarettes typically ranges from 1 to 2 μg/cigarette [50] and, the average blood cadmium concentration for the general non-smoking population with non-occupational exposure rarely exceeds 8.9 nmol/L (1 μg/L) [51]. In Nunavik, the prevalence of cigarette smoking is very high (smokers represent approximately 70% of the adult population) [52]. According to recent studies, mean blood cadmium concentrations in the smoking population of Quebec have decreased in the last 10 years [53], which suggests that smoking habits and/or cadmium content of cigarette may have changed.

In 1992, Santé Québec conducted the first major health survey to assess the general health status of the Inuit population of Nunavik, and mercury, lead and cadmium concentrations were high compared to the concentrations measured in the general population of Canada [5,48]. Given the known toxic effects of these metals in adults and the developmental effects observed following *in utero *exposure, the objective of this study was therefore to evaluate blood concentration of mercury, lead and cadmium and its sources among the Inuit of Nunavik in 2004 and evaluate the evolution of theses concentrations since the Santé Québec Survey in 1992 [52].

## Methods

### Population and sampling

**Figure 1:**
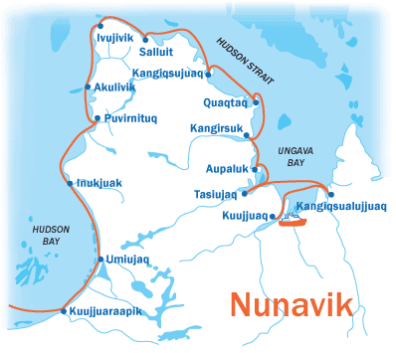
The Amundsen's route around Nunavik.

The Nunavik Health Survey [52] was conducted in the 14 communities of Nunavik during fall 2004 on the Canadian Coast Guard icebreaker and scientific research vessel *CCGS Amundsen *(Fig. 1), in collaboration with the *Institut National de Santé Publique du Québec*, the Nunavik Regional Board of Health and Social Services and the Institut de la Statistique du Québec (ISQ). The ISQ was given the mandate to develop the survey frame. Many sources of information were used by the ISQ to count all private Inuit households in Nunavik. Priority was given to municipal rolls as the most comprehensive source of information. When information was lacking, other lists were used such as those from employers who provide lodging to employees, (Ungava's Tulattavik Health Centre, Inuulitsivik Health Centre, Kativik School Board, etc.), the Québec electoral roll, the Kativik Housing Bureau and the telephone directory.

A stratified random sample of private Inuit households was selected according to the number of residents per municipalities. Since home addresses in some municipalities are consecutive, the survey frame was sorted first by home addresses, followed by a systematic draw of a predetermined number of households to avoid selection of two immediate neighbours. Since many Inuit regularly move from one house to another, it was decided to sample households instead of individuals. To obtain a good representation of each community, a proportional allocation of sample units corresponding to the size of each village was chosen. All eligible people of the household were asked to participate according to the survey steps or instruments. A total of 1056 people were recruited for the study and informed consent was obtained from all participants. Blood samples were obtained from 917 participants during the clinical session to evaluate levels of exposure to environmental contaminants. Face-to-face interviews were conducted on board the *Amundsen *to collect information on socio-demographic characteristics and lifestyle habits. A food frequency questionnaire was administered to collect information on food intakes and eating patterns. The questionnaire used in 2004 measured the consumption of 25 food items of *country foods *which refers to food items derived from fishing, hunting and gathering, recorded for each of the four seasons (of the year prior the interview). Specification of the usual serving size was included in the questions on frequency. Pre-defined serving sizes were included in the questionnaire and a corresponding food model was shown to the respondents. The study protocol was approved by the Ethics Committee of Laval University and Public Health Ethics Committee of Québec.

To assess temporal variation of blood mercury, lead and cadmium concentrations, we compared our results with data from a survey conducted within the same population with a similar protocol by Santé Québec in 1992 [5,48,52]. For more detailed information on the methodology, please refer to theses publications. A systematic sampling was achieved after sorting the survey base by household address to favour a more complete coverage of the territory and to avoid the selection of next-door neighbours. Furthermore, so that each village would be represented, the sample was stratified by village, with quasi-proportional representation of the number of households in each stratum. A total of 400 households were randomly selected and visited by interviewers between September and December 1992, and a total of 493 people were recruited. Protocols for face-to-face interviews and blood sampling were similar to the ones used in 2004. However, the food frequency questionnaire was only administered to 226 women, and the list of country foods was les exhaustive than that used in 2004, hence making comparisons between the surveys impossible with regards to these predictors.

### Biological samples processing

The blood sample for metal analysis was collected from a cubital vein in a 6-mL plastic vacutainer containing potassium EDTA as the anticoagulant (BD Medical). Once collected, blood samples were kept at 4°C until stored at -20°C on the ship. During their transfer from the ship to Québec City, the samples were kept frozen in insulated containers with ice packs. Laboratory analysis for contaminants was performed at the *Centre de Toxicologie *of *the Institut National de Santé Publique du Québec*. This facility is accredited ISO 17025 and participates in the QA/QC program of the Canadian Northern Contaminants Program and the Arctic Monitoring Assessment Program.

In 2004, total mercury, lead and cadmium were determined in whole blood samples from individual participants by inductively coupled plasma mass spectrometry (ICP-MS), which allows the simultaneous determinations of several metals in elementary form in various matrices. Blood samples are diluted in ammonium hydroxide and metals are brought to their elementary form by passing through argon plasma before being identified by mass spectrometry. All samples were analysed on Perkin Elmer Sciex ICP-MS instruments: lead and cadmium were quantified on the Elan 6000 and total mercury on the DRC II. Detection limits were 1 nmol/L for lead (1 μmol = 207.2 μg), 0.4 nmol/L for cadmium (1 nmol = 0.11 μg) and 0.5 nmol/L for total mercury (1 nmol = 0.20 μg). Accuracy and precision were measured using reference material from the Interlaboratory Comparison Program of the *Centre de Toxicologie* (INSPQ). The coefficient of variation was 2.8%, and the relative bias was +3.1% for the lead reference specimen analysed on 10 different days (consensus median value from participating laboratories = 0.6 μmol/L). The coefficient of variation was 7.2%, and the relative bias was 0.6% for the cadmium reference specimen (39 nmol/L; n = 10). The coefficient of variation was 2.1%, and the relative bias was +1.6% for the total mercury reference specimen analysed on 10 different days (consensus median value from participating laboratories = 66 nmol/L).

In 1992 blood lead and cadmium concentrations were determined by graphite furnace atomic absorption spectrometry (GFAAS) (Perkin Elmer, model ZL 4100). Samples were diluted and injected directly into the instrument. Blood total mercury concentrations were determined by cold-vapor atomic absorption spectrometry (CVAAS) (Pharmacia Mercury monitor). Samples were microwave-digested with nitric acid, and an aliquot was used for the analysis. In order to compare the different laboratory procedures used in 1992 and 2004, the INSPQ performed a quality controle study within the framework of the Québec Interlaboratory Comparison Program. The INSPQ used both methods to determine the total mercury, lead and cadmium concentrations and obtained strong correlations. The biais observed was 8% for total mercury (y = 0.92x + 2.83 ; R^2 ^= 0.99), 6% for lead (y = 0.94x - 0.0025 ; R^2 ^= 0.93) and 1% for total mercury (y = 0.99x + 1.80 ; R^2 ^= 0.98),. We can therefore assume that both methods (ICP-MS and furnace atomic absorption spectrometry) give comparable results.

### Statistical analysis

Descriptive statistics were performed in order to present consumption of country food items and metals concentrations in whole blood. Blood mercury and lead concentrations as well as the most consumed food items (marine mammal meat, fish, terrestrial mammal meat and game birds meat) satisfied log-normality criteria and geometric mean were therefore used. Cadmium concentrations did not satisfy the normality criteria due to the strong influence of tobacco consumption. However, once adjusted for tobacco consumption (smoker, ex-smoker, never-smoker), the cadmium distribution was log-normal therefore, geometric mean was used as the measure of central tendency, and smoking habits were forced in the regression models. Student's t-tests or analyses of variance were used to compare variables according to gender, age category, region of residence, and smoking habits. For the purpose of this study, the Nunavik territory was divided in two regions (Hudson Bay and Ungava Bay regions) in relation to the different dietary habits of the residents. Other country food items were consumed by less then 50% of the sample, therefore these variables were treated dichotomously (consumer, non-consumer).

Chi-square test was use to compare proportions and Mantel-Haenszel Chi-square test was use to evaluate trend. Comparisons have also been made with data obtained from the 1992 Santé Québec Survey. The variation in metal concentrations between 1992 and 2004, stratified according to gender, age category, region of residence and tobacco consumption was compared using Students't-tests. We performed analyses of variance to assess multivariate associations between metal blood concentrations and various variables. All personal characteristics (gender, age, region of residency) and consumption habits that were associated (p ≤ 0.10) with metal concentrations in blood were considered in the predictive model. To be retained in the final model, a variable had to show significant association (i.e., p ≤ 0.05) with metal concentrations in blood.

Given the complex sampling procedures used in both surveys, all analyses were weighted to take into account the population's structure with regards to municipality, gender and age. Adjustment for age is made on the basis of 5-year age categories using Nunavik 2001 census of Statistics Canada as reference population. However, only raw data is reported in the text, tables and figures to avoid any possible confusion with adjusted proportions. All analyses of variance used a Satterthwaite correction to account for the sampling strategy used in both surveys and the results are presented as Satterthwaite chi-squared. Statistical analyses were conducted using the SAS statistical Package v. 9.1 (SAS Version 9.1, SAS Institute, Cary, NC) with an α threshold of 0.05.

## Results

Table 1 shows that the 917 participants of the 2004 survey and the 493 participants of the 1992 survey had similar characteristics and then, results obtained from those two studies were comparable. Men and women were equally represented in 2004 and had the same age structure (results not shown). The mean age for men in 2004 was 36.4-years old (95% confidence interval (CI):35.9, 37.0) and 36.9-years old for women (95%CI: 36.2, 37.2). In 2004, smokers represent 77.5% of the sample.

**Table 1:** Distribution of participants by gender, age, smoking status, region of residence and municipality (1992, 2004)

	**1992 (n = 493)**		**2004 (n = 917)**	
	N^1^	%^2^	N^1^	%^2^

**Gender**				
Men	209	51.2	414	51.5
Women	284	48.9	503	48.5
**Age groups**				
18–24	107	27.8	206	23.9
25–44	233	47.7	471	50.4
45–74	153	24.5	240	25.7
**Smoking habits**				
Non smokers	42	10.0	76	8.9
Ex-smokers	79	16.3	119	13.6
Smokers	312	73.8	663	77.5
**Region of residence**				
Hudson Bay	274	59.1	497	56.7
Ungava Bay	219	40.9	420	43.3

^1 ^Crude sample size

^2 ^Weighted values

Regarding smoking habits (results not shown), results from 2004 showed that tobacco use (smokers, ex-smokers, never-smokers) decreased significantly with age group (χ^2 ^= 101.9; p < 0.0001); 92.1% of the 18–24 year old group were smokers and 57.2% of the 45–74-year old group, but this trend only showed borderline significance (*p *trend = 0.06). Smoking habits were not significantly different according to gender (χ^2 ^= 6.0; p = 0.2). The proportion of smokers was significantly higher in Hudson Bay residents (χ^2 ^= 10.2; p = 0.04) than in Ungava Bay residents

The mean daily consumption of the most consumed country food items is presented in table 2. Only consumption of fish significantly increased with age (p = 0.0001). Consumption of marine mammal meat (p < 0.0001), fish (p = 0.01) and game birds meat (p = 0.01) was significantly higher in men than women. In Hudson Bay region, mean consumption of marine mammal meat (p < 0.0001), fish (p = 0.04), and game birds meat (p = 0.01) was significantly higher than in Ungava Bay. Other country food items were consumed by less then 50% of the sample, therefore these variables were treated dichotomously, and proportions of consumers are presented in table 3. Because only 5% (n = 37) of the sample were consumers of caribou liver and kidney, repartition of the sample according to age and gender is not presented for this food item. Proportion of consumers of marine mammal kidneys and liver (p-trend = 0.003), salmon and trout (p-trend <0.0001), duck meat (p-trend <0.0001) and caribou liver and kidney (p-trend = 0.02) increased significantly with age. Proportion of consumers did not vary according to gender, except for consumption of salmon and trout (χ^2 ^= 12,6; p < 0.001). In Hudson Bay region, the proportion of consumer of marine mammal kidneys and liver (χ^2 ^= 17.7; p < 0,0001) and duck meat (χ^2 ^= 40.7; p < 0,0001) were higher than in Ungava Bay, while it was lower for consumption of salmon and trout (χ^2 ^= 41.7; p < 0,0001) and caribou liver and kidney (χ^2 ^= 16.1; p < 0,0001) (results not shown).

**Table 2:** Mean daily consumption of various country food items by gender and age, 2004

	**Men**			**Women**		
	
	**N**	**Geometric Mean**	**95% CI**	**N**	**Geometric Mean**	**95% CI**
**Marine mammal meat **^1^						
Age group 18–24	76	3.1	2.2–4.5	92	2.4	1.9–3.1
25–44	184	3.8	3.0–4.7	212	2.1	1.7–2.5
45–74	79	4.4	3.2–5.8	105	2,7	2.1–3.6
All	339	3.8	3.2–4.4	409	2.3	2.0–2.6
**Fish**^1^						
Age group 18–24	78	17.3	12.1–24.7	94	13.0	9.2–18.4
25–44	189	29.3	24.0–36.0	218	22.0	18.2–26.6
45–74	87	37.7	28.8–49.4	110	28.1	21.6–36.5
All	354	27.5	23.6–32.1	422	20.9	18.2–24.0
**Terrestrial mammal meat**^1^						
Age group 18–24	78	17.1	12.0–24.2	94	19.6	14.2–26.9
25–44	189	23.9	19.5–29.4	215	20.4	17.2–24.2
45–74	85	26.4	20.4–34.2	108	19.1	14.2–25.5
All	352	22.6	19.4–26.3	417	19.8	17.4–22.6
**Game birds meat**^1^						
Age group 18–24	78	12.8	9.2–17.9	94	6.1	4.3–8.8
25–44	189	10.3	8.1–13.1	218	8.1	6.6–10.0
45–74	87	13.5	9.8–18.6	111	10.0	7.4–13.5
All	354	11.6	9.8–13.8	422	8.1	6.9–9.4

^1 ^Grams per day, annual basis

**Table 3:** Proportion of consumers of various country food items by gender and age, 2004

	**Men**		**Women**		**All**	
	**N**^**2**^	**%**^**3**^	**N**^**2**^	**%**^**3**^	**N**^**2**^	**%**^**3**^

**Marine mammal kidneys and liver**^1^						
Age group 18–24	25	30.0	22	21.7	47	26.2
25–44	58	33.6	70	35.7	128	34.6
45–74	35	47.8	94	34.4	67	41.4
All	118	36.1	124	32.0	242	34.2
**Salmon and trout**^1^						
Age group 18–24	31	39.0	21	19.6	52	30.1
25–44	11	59.6	94	43.7	205	52.2
45–74	54	64.1	73	66.7	127	65.4
All	196	55.9	188	44.6	384	50.5
**Duck meat**^1^						
Age group 18–24	7	10.9	9	8.8	16	9.9
25–44	38	20.7	27	13.1	65	17.1
45–74	31	35.5	40	36.6	71	36.0
All	76	22.1	76	18.6	152	20.4

^1 ^Consumption is defined as 'at least once a year'

^2 ^Crude sample size

^3 ^Weighted values

**Figure 2:**
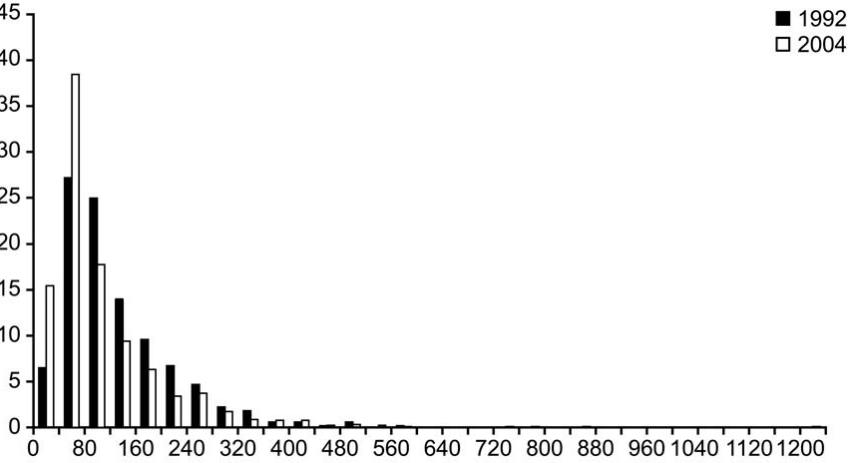
Frequency distribution of mercury concentrations (nmol/l) in blood samples of 917 Inuits from Nunavik.

**Figure 3:**
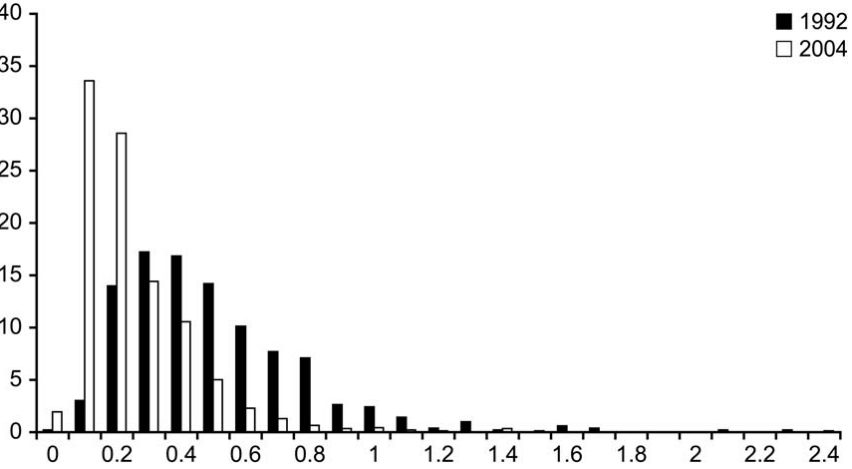
Frequency distribution of lead concentrations (μmol/l) in blood samples of 917 Inuits from Nunavik.

**Figure 4:**
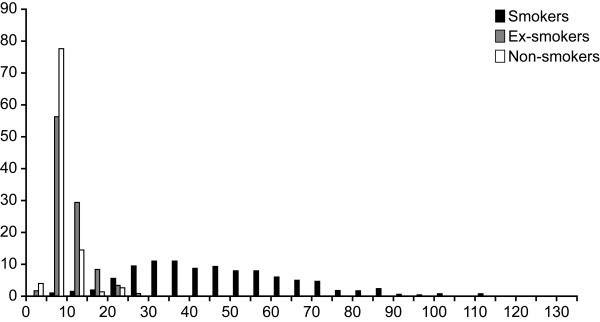
Frequency distribution of cadmium concentrations (nmol/l) in blood samples of 917 Inuits from Nunavik, 2004.

Figure 2, 3 and 4 present the distribution of the mercury, lead and cadmium (according to smoking status) concentrations measured in blood of Inuit adults in 2004. These metals were detected in all 917 samples. Table 4 presents descriptive statistics for the blood concentrations of metals detected among the Inuit adult population aged 18 to 74 during the 1992 Santé Québec Survey and the 2004 Nunavik Inuit Health Survey, stratified for gender, age and region of residence, respectively. In 2004, mean blood mercury concentrations were significantly higher in women than in men, whereas mean lead concentrations were higher in men and cadmium concentrations did not vary significantly according to gender. Adults aged 45 to 74 had significantly higher concentrations (p < 0.001) of mercury and lead than younger adults. Blood cadmium concentrations decreased with age categories (p < 0.001), with a geometric mean from 37.7 nmol/l in 18–24-year age group to 17.6 nmol/L in 45–74-year age group. However, after adjusting for smoking and its interaction with age (results not shown), logged cadmium concentrations were not significantly different according to age (p = 0.4). Mean mercury and cadmium concentrations were significantly higher in communities along Hudson Bay (p < 0.001), but no difference was observed between the two regions for blood lead concentrations (p = 0.19).

**Table 4:** Blood mercury, lead and cadmium concentrations by gender, age and residence (1992, 2004)

	**1992**			**2004**				
	**N **^1^	**Geometric Mean**	**95% CI**	**N **^1^	**Geometric Mean**	**95% CI**	**Range**	**P-value **^2^

**Mercury (nmol/L)**								

All	492	74.8	(69.2–80.9)	917	51.2	(47,9–54,6)**	0.4–1200	
Gender								
Men	209	70.3	(62.1–79.6)	414	45.8	(41.5–50.5)**	0.4–1200	< 0,001
Women	283	79.9	(74.4–85.8)	503	57.6	(53.7–61.8)**	1.0–820	
Age groups								
18 to 24 years	107	50.6	(43.0–59.7)	206	31.5	(27.7–35.8)**	2.2–820	< 0.001
25 to 44 years	233	69.2	(62.3–76.7)	471	44.3	(40.1–48.9)**	0.4–420	
45 to 74 years	152	135.9	(120.2–153.6)	240	106.6	(96.1–118.2)*	4.6–1200	
Women of childbearing age (18 to 39 years)	175	64.5	(59.2–70.3)	308	41.7	(38.2–45.6)**	1.0–820	
Region of residence								
Hudson Bay	274	93.1	(84.2–102.8)	497	58.7	(53.1–64.8)**	0.4–1200	< 0.001
Ungava Bay	218	54.6	(48.4–61.6)	420	42.8	(38.4–97.1)**	1.0–520	

**Lead (μmol/L)**								

All	493	0.42	(0.40–0.44)	917	0.19	(0.18–0.20)**	0.028–2. 4	
Gender								
Men	209	0.46	(0.43–0.49)	414	0.22	(0.21–0.24)**	0.044–2.4	< 0,001
Women	284	0.38	(0.35–0.40)	503	0.17	(0.16–0.17)**	0.028–1.5	
Age groups								
18–24	107	0.31	(0.28–0.34)	206	0.14	(0.13–0.15)*	0.033–0.8	< 0.001
25–44	233	0.43	(0.40–0.46)	471	0.19	(0.17–0.20)*	0.028–2.4	
45–74	153	0.56	(0.52–0.60)	240	0.29	(0.27–0.31)*	0.039–1.5	
Women of childbearing age (18 to 39 years)	175	0.33	(0.31–0.36)	308	0.13	(0.12–0.14)*	0.028–1.0	
Region of residence								
Hudson Bay	274	0.48	(0.44–0.51)	497	0.20	(0.19–0.21)*	0.036–2.4	0.19
Ungava Bay	219	0.35	(0.33–0.37)	420	0.19	(0.18–0.20)**	0.028–1.4	

**Cadmium (nmol/L)**								

All	493	33.2	30.7–35.9	917	26.0	24.3–27.8**	1.4–130	
Gender								
Men	209	32.3	28.4–36.8	414	25.5	23.0–28.3*	2.1–110	0.6
Women	284	34.1	31.0–37.6	503	26.5	24.3–28.9*	1.4–130	
Age groups								
18–24	107	31.0	25.7–37.5	206	37.7	33.4–42.5	2.1–110	< 0.001
25–44	233	37.7	34.0–41.7	471	27.7	25.320.0**	1.4–110	
45–74	153	28.0	24.2–32.5	240	17.6	19.5–20.0**	2.0–130	
Women of childbearing age (18 to 39 years	175	46.7	42.9–50.5	308	38.1	36.0–40.3	1.4–110	
Residence								
Hudson Bay	274	35.4	31.8–39.4	497	28.7*	26.3–31.4	2.0–110	< 0.001
Ungava Bay	219	30.2	26.9–33.9	420	22.8*	20.5–25.2	1.4–130	
Smoking status								
Never Smoker	42	7.7	6.4–9.2	76	5.3 **	4.7–5.8	1.4–22	< 0.001
Ex Smoker	79	12.7	11.1–14.4	119	6.6**	6.0–7.3	2.0–23	
Smoker	312	50.2	47.6–52.3	663	40.4**	38.9–42.1	3.2–130	

^1 ^Crude sample size

^2 ^P-value for comparison between categories in 2004; based on the Satterthwaite χ^2 ^test

* Significant difference between health surveys p < 0.01

** Significant difference between health surveys p < 0.001

Analyses of variance (results not shown) also showed that mercury concentrations were not significantly different between smokers and never-smokers (p = 0.63), and that lead concentrations were associated with hunting frequency, but not with alcohol consumption (p = 0.12) and tobacco consumption (p = 0.36). Cadmium concentrations significantly decreased according to BMI (p < 0.001) (results not shown). Higher cadmium concentrations were observed in smokers (7.6 fold), compared with ex-smokers and never-smokers (p < 0.001), and concentrations significantly increased with the number of cigarettes smoked per day (p < 0.001) (results not shown). Blood cadmium concentrations were higher among consumers of caribou kidneys and liver (geometric mean of 32.3 nmol/l for consumers and 26.3 nmol/l for non-consumers) but this association became non significant after controlling for tobacco consumption and other confounders, due to the strong impact of smoking (results not shown). When we limited our analyses to non-smokers, mean cadmium concentration was significantly higher among consumers of caribou kidneys and liver, even after controlling for region of residence (p = 0.03).

As shown in Table 4, statistically significant declines (p < 0.001) in mercury (32%), lead (55%) and cadmium (22%) exposures were observed between 1992 and 2004, and this was observed in both genders. Metals concentrations also decreased for all age categories, and for both regions of residence, between 1992 and 2004.

A positive association (Table 5) was observed between blood mercury concentrations and consumption of marine mammal meat, marine mammal kidney and liver and salmon and trout. A model including consumption of marine mammal meat, kidney and liver, age, gender and region of residence as independent variables explained 27% of the variation of mercury concentration (p < 0.0001). Of all theses variables, age was the most highly associated to mercury blood concentrations (partial R^2 ^of 0.20), followed by marine mammal meat consumption (partial R^2 ^of 0.04). As shown in Table 5, all the traditional food consumption variables selected were correlated with lead concentrations. A model including consumption of game birds and marine mammal kidneys and liver, age, gender and tobacco consumption as independent variables explained 25% of the variation of lead concentration (p < 0.0001). However, associations with dietary variables were very low (partial R^2 ^of 0.01) and as for mercury, age was the most highly associated variable (partial R^2 ^of 0.20). Finally, a multivariate regression model including smoking status, region of residence and BMI explained 73% of the blood cadmium variation. Smoking status was by far the most important predictor (partial R^2^:=0.56; p < 0.0001).

**Table 5:** Predictive models for blood mercury, lead and cadmium concentrations, 2004

**Model**	**Beta**	**Partial R^2^**	**P value**
**Mercury (log values) (R**^**2**^**= 0.27, p < 0.0001)**			
Consumption of marine mammal meat^1^	0.07	0.04	< 0.0001
Consumption of marine mammal kidneys and liver: Consumers	0.23	0.01	0.003
Age (years)	0.03	0.20	< 0.0001
Gender: Men	- 0.24	0.02	0.001
Region of residence : Hudson Bay	0.24	0.02	0.001

**Lead (log values) (R^2 ^= 0.25, p < 0.0001)**			
Consumption of game birds^1^	0.001	0.01	0.002
Consumption of marine mammal kidneys and liver: Consumers	0.11	0.01	0.03
Age (years)	0.02	0.20	< 0.0001
Gender: Men	0.28	0.04	< 0.0001
Smoking status : Smoker	0.21	0.04	< 0.0001

**Cadmium (Log values) (R^2 ^= 0.73, p < 0.0001)**			
Smoking status : Smoker	1.99	0.56	< 0.0001
Ex-smoker	0.23	0.01	0.004
Region of residence : Hudson Bay	0.10	0.01	0.01
BMI (kg/m^2^)	- 0.01	0.02	< 0.0001

^1 ^Grams per day, annual basis

## Discussion

This study aimed at describing current exposure to mercury, lead and cadmium in the Inuit population of Nunavik, identify current dietary sources of exposure and compare these levels to those prevailing 12 years ago. The results showed a general decrease in exposure to these metals during the 12 year time span.

### Mercury

As in 1992, average blood mercury concentrations in 2004 were statistically higher in women than in men. The associations between mercury and gender reported in other studies vary according to different populations [54,55]. Mercury blood concentrations were statistically higher in adults aged 45 to 74 compared to younger adults, as observed in 1992 and in other studies [4,54,56,57]. Since mercury is not known to bioaccumulate in human tissues, this association with age could reflect the tendency of young adults to eat less traditional food (other than marine mammal meat, liver and kidneys) than the young generation. Indeed, we have observed a significant lower intake of certain traditional food items containing mercury (fish, duck meat) in younger people.

As in 1992 [5], blood concentrations of mercury were higher in Hudson Bay residents compared to Ungava Bay residents. A possible explanation for this difference is that Hudson Bay residents consume significantly more marine mammal meat, kidney and liver, which are known sources of mercury intake [58-60]. Indeed, in the present study, blood concentrations of mercury were mainly explained by age (partial R^2^: 0.20) and consumption of marine mammal meat (partial R^2^: 0.04), liver and kidney (partial R^2^: 0.01). These observations match the results obtained in 1992, where mercury concentrations were correlated with age and consumption of beluga and seal meat and liver (R^2^: 0,30) [5].

Between the 1992 and 2004 health surveys, an important decrease (32%) in blood concentrations of mercury was observed in the Inuit population of Nunavik. However, levels of mercury in the environment do not seem to have shown a consistent decrease over the last decade [61,62]; on the contrary, evidence for increasing levels of mercury in the Canadian Arctic is observed in a number of marine birds and mammals [63-66]. It is therefore more likely that the decrease observed in blood concentrations of mercury in Nunavik Inuit could be attributed to changes in dietary habits. Indeed, the mean intake of marine mammal meat decreased from 28.7 g/day in 1992 to 17.5 g/day in 2004, which represents an approximate 40% decrease [67]. These changes in dietary habits could result from the promotion of less contaminated traditional food (such as Arctic Char) in the Arctic, the decrease in traditional food consumption associated to a shift to a globally more westernized life-style. Despite the observed decrease, mercury body burden in this population nevertheless remains a concern, based on the proportion of the population showing blood concentrations above the maximum recommended level.

Mean blood mercury concentrations (51.2 nmol/L) was still much higher in Nunavik than in the general population of Québec City (3.7 nmol/L;n = 470) [53]. This mean concentration was were also higher than those observed in the Cree populations of Oujé-Bougoumou (21.3 nmol/L; n = 169) and Nemaska (14.4 nmol/L; n = 71) (province of Québec, Canada) [68]. However, mean blood mercury concentration in Nunavik was much lower than those recently measured in specific groups such as high-end fish consumers from San Francisco [69], fish consumers from Brazilian Amazon [70] and Inuit population from Greenland [71].

Mean blood mercury concentration (51.2 nmol/L) was lower than the acceptable blood concentration of 99.7 nmol/L established by Health Canada for the general adult population [72], but the maximum concentration reached 1200 nmol/L, which is 12 times higher then the maximum recommended concentration. Furthermore, 28% of individuals from the general population of Nunavik and 72% of women of reproductive age were above their respective recommended blood level (99.7 nmol/L for the general population; 28.9 nmol/L women of childbearing age).

### Lead

Mean blood concentrations of lead were higher in men and in adults aged 45 to 74, a finding that is consistent with data from 1992 and other studies [4,5,73-75]. Milman et *al *(1994) suggest that the gender difference observed in lead body burdens could be explained by the higher content of haemoglobin in men's blood [56]. Reinforcing this possible explanation for gender difference, 40% of women in Nunavik were anemic in 2004 [76]. Blood lead concentrations increased slightly with increasing annual game bird consumption, a finding which is again consistent results from 1992 and other studies [5,22,73,77]. A significant association could be observed with smoking, as reported in 1992 and in other studies [5,6,21,57].

Blood lead concentrations showed a 55% decrease over the 12-year period. This decreasing trend has also been observed in cord blood obtained from Nunavik newborns, with markedly lower concentrations in 1999 [23]. This suggest that the strong decrease in lead concentrations in adults and newborns could be not only attributed to the decreasing environmental levels (since the ban of leaded gasoline), but could also be a beneficial consequence of the ban on lead shot use for hunting wild game and birds, a policy implemented by the Public Health Directorate in 1998 [21]. In an effort to reduce exposure the public health authorities of Nunavik also actively informed the population about the toxic effects of lead from ammunition on children's health [21]. However, mean blood concentration of lead observed in 2004 (0.19 μmol/l) was still higher than the concentrations observed in the general population of southern Québec (0.10 μmol/L; n = 441) [53], as well as levels measured in the native Cree populations of Oujé-Bougoumou (0.10 μmol/L; n = 169) and Nemaska (0.10 μmol/L; n = 71) [78].

Mean blood lead concentrations was lower than the maximum concentration recommended by Health Canada (0.48 μmol/L) [79], but still, almost 10% of the population sample and 2% of women of childbearing age showed levels above the maximum recommended level, with a maximum observed of 2.4 μmol/L.

### Cadmium

Cadmium concentrations were higher in Hudson Bay than in Ungava Bay region, increased with smoking intensity (cigarettes/day) and decreased with BMI (kg/m^3^). Blood cadmium concentrations are often higher in women than in men, in never-smokers, because of a higher gastrointestinal absorption [80], generally linked to an iron deficiency. In our 2004 population sample, concentrations were higher in women but this gender difference was not significant, contrary to data reported in other studies [56,81]. Cadmium accumulates in the kidneys over life time, but blood concentrations reflect mainly recent exposure, while urine concentrations reflect both recent and cumulative exposure (kidney cortex concentrations). Therefore we did not observed increasing blood cadmium concentrations with age but rather decreasing concentrations, which could be explained by the decreasing proportion of smokers observed with groups of increasing age (85% in the 18–24-year age group, 76% in the 25-year age group, and 53% in the over 45-year age group). Indeed, after adjusting for smoking and its interaction with age, this decrease was not significant

Since the Santé Québec Health Survey of 1992, blood cadmium concentrations have globally decreased by 22% in the Inuit population. The most important decrease of cadmium blood concentrations was observed among ex-smokers (48%), followed by never-smokers (31%), compared to smokers (20%). A decrease has also been observed, to a more extent, for the general population of the Province of Québec, blood cadmium concentrations in smokers falling from 46.1 nmol/L in 1994 [47] to 31.1 nmol/L in 2001 (decrease of 32%) [82], and therefore, we suggest that it could be related to a decrease in the cadmium content of cigarette tobacco, since smoking is by far the main source of cadmium exposure in both populations. The important decrease in never-smokers and ex-smokers could be explained by a decrease in passive tobacco smoke exposure following preventive campaigns promoting smoke-free environments. Although studies carried out in 1988, 1990 and 1992 failed to show a significant association between consumption of caribou liver and kidneys [46-48] and cadmium exposure, our results revealed that dietary habits nevertheless have an impact on blood cadmium concentrations in never-smokers and therefore, the effect of the consumption of caribou offal cannot be completely dismissed as an additional source of exposure. Overall, the consumption of caribou liver and kidneys constitutes only a small proportion of the traditional Inuit diet and it is unlikely that it could ever lead an individual to exposure levels exceeding safety thresholds [44], and therefore, environmental tobacco smoke definitely remains the main source of cadmium exposure as shown by the multivariate regression model.

Regardless of the observed decrease in exposure, a third of the sample still displayed blood concentrations exceeding maximum concentration of 44.5 nmol/L recommended by several international health authorities [83,84], with concentrations reaching a maximum of 130 nmol/L. All individuals showing concentrations above the recommendation were current smokers, and among daily smokers, the proportion of individuals exceeding the recommendation increased with the number of cigarettes smoked per day.

## Conclusion

These encouraging results demonstrate that implementation of public health campaigns, such as the ban of lead cartridges, may reduce Inuit exposure to toxic metals. For mercury, important decreases in blood concentrations were observed and could be explained by dietary changes. Nevertheless, a significant proportion of the population still show blood concentrations above the maximum recommended levels. Promoting the consumption of less contaminated seafood should therefore continue, especially for more sensitive populations such as pregnant women and women of childbearing age. Results on cadmium exposition underscore the need for preventive actions to be taken and for existing campaigns and actions to be re-enforced in order to decrease the proportion of smokers in the Nunavik population, especially in younger age groups where smoking is more frequent and has a stronger potential for developing adverse long-term health effects

## Competing interests

The authors declare that they have no competing interests.

## Authors' contributions

JF performed the statistical analyses, interpretation of data and wrote the manuscript.

ED conceived of the study, participated in its design and coordination and helped writing the manuscript.

PA, DP and SD participated in the design of the study, provided support in the interpretation of data, and manuscript writing.

All authors have read and approved the final version of the manuscript.
